# Effect of grain boundary resistance on the ionic conductivity of amorphous *x*Li_2_S-(100-*x*)LiI binary system

**DOI:** 10.3389/fchem.2023.1230187

**Published:** 2023-07-20

**Authors:** Longbang Di, Jiangyang Pan, Lei Gao, Jinlong Zhu, Liping Wang, Xiaomeng Wang, Qinqin Su, Song Gao, Ruqiang Zou, Yusheng Zhao, Songbai Han

**Affiliations:** ^1^ Academy for Advanced Interdisciplinary Studies, Southern University of Science and Technology, Shenzhen, China; ^2^ School of Materials Science and Engineering, Peking University, Beijing, China; ^3^ Eastern Institute for Advanced Study, Ningbo, China

**Keywords:** solid-state electrolytes, ionic conductivity, amorphous, grain boundary resistance, mechanochemical milling

## Abstract

Solid-state electrolytes (SSEs) hold the key position in the progress of cutting-edge all-solid-state batteries (ASSBs). The ionic conductivity of solid-state electrolytes is linked to the presence of both amorphous and crystalline phases. This study employs the synthesis method of mechanochemical milling on binary *x*Li_2_S-(100-*x*)LiI system to investigate the effect of amorphization on its ionic conductivity. Powder X-ray diffraction (PXRD) shows that the stoichiometry of Li_2_S and LiI has a significant impact on the amorphization of *x*Li_2_S-(100-*x*)LiI system. Furthermore, the analysis of electrochemical impedance spectroscopy (EIS) indicates that the amorphization of *x*Li_2_S-(100-*x*)LiI system is strongly correlated with its ionic conductivity, which is primarily attributed to the effect of grain boundary resistance. These findings uncover the latent connections between amorphization, grain boundary resistance, and ionic conductivity, offering insight into the design of innovative amorphous SSEs.

## 1 Introduction

All-solid-state batteries (ASSBs) offer a viable solution to mitigate the safety concerns of conventional lithium-ions batteries (LIBs), in addition to their potential for exploiting the Li-metal anode with a theoretical specific capacity of 3,860 mAh g^−1^ and electrochemical potential of −3.04 V *versus* the standard hydrogen electrode, thereby enabling a significant enhancement of the energy-density of the batteries (Janek and Zeier, 2016). To make ASSBs practical, it is crucial to advance the development of solid-state electrolytes (SSEs) with exceptional performance (Xia et al., 2019; Abakumov et al., 2020; Zhao et al., 2020). Typical SSEs mainly include sulfide, halide, oxide, and other systems. Sulfide SSEs commonly exhibit high ionic conductivity and good processability, but the low intrinsic electrochemical stability windows (Zhu et al., 2015). Halide SSEs offer high ionic conductivity and compatibility with high voltage cathodes such as LiCoO_2_, but are not stable with Li-metal anode (Kwak et al., 2022). Oxide SSEs exhibit wide electrochemical stability windows, but feature high interfacial and grain boundary resistance (van den Broek et al., 2016). Each SSE owns distinct properties in terms of ionic conductivity, electrochemical window, and stability in the air (Kamaya et al., 2011; Manthiram et al., 2017; Yao et al., 2019; Kim et al., 2020; Li et al., 2020; Xu et al., 2023). Notably, ionic conductivity is a vital performance indicator that impacts the application of SSEs (Kwak et al., 2022; Yang and Wu, 2022).

The ionic conductivity of SSEs can be optimized by manipulating lattice structure, element substitution, phase change, amorphization, etc (Asano et al., 2018; Wang et al., 2019; Luo et al., 2021; Kwak et al., 2022; Schweiger et al., 2022; Szczuka et al., 2022). Among these methods, amorphization has gained attention due to the emergence of mechanochemical synthesis methods, which is an effective approach to synthesizing SSEs with lower grain boundary resistance (Dalvi and Shahi, 2004; Morimoto et al., 2004; Kim and Martin, 2006; Enayati and Mohamed, 2014). Representatively, the amorphous Li_2_S-P_2_S_5_ binary system SSEs can be prepared by mechanical milling and exhibit a high ionic conductivity (>10^–4^ S/cm) (Hayashi et al., 2004). In addition, some SSEs such as Li_6_PS_5_I (Brinek et al., 2020), Li_2_B_4_O_7_ (Wohlmuth et al., 2016), Li_2_ZrCl_6_ (Chen et al., 2021), and Li_3_YCl_6_ (Asano et al., 2018) show higher ionic conductivity after undergoing amorphization. However, the impact of amorphization on the ionic conductivity varies depending on the specific SSEs system, crystalline structures play a critical role in ionic conductivity for numerous SSEs. (Zhao et al., 2019; Schweiger et al., 2022). For instance, a recent study by Schweiger et al. revealed that Li_10_GeP_2_S_12_ experienced an increase in grain boundary resistance and a decrease in ionic conductivity with increasing milling time against the behavior of other SSEs. The mechanism behind this phenomenon is that defects and site disorder caused by ball milling impede the migration of lithium ions within the lattice (Schweiger et al., 2022). Therefore, it is essential to investigate the impact of amorphization on the grain boundary resistance and ionic conductivity of SSEs, while also elucidating the underlying mechanism.

In this study, the amorphous SSEs of binary *x*Li_2_S-(100-*x*)LiI (10 ≤ *x* ≤ 90) were synthesized by mechanical ball-milling method for the first time. PXRD analysis indicates that the amorphization degree of *x*Li_2_S-(100-*x*)LiI system is significantly influenced by the stoichiometry of Li_2_S and LiI. Furthermore, electrochemical impedance spectroscopy (EIS) analysis reveals a strong correlation between the amorphization degree of the *x*Li_2_S-(100-*x*)LiI system and its ionic conductivity, with the effect of grain boundary resistance being the primary contributing factor. Additionally, the increase of Li_2_S content in *x*Li_2_S-(100-*x*)LiI may restrict the grain boundary impedance reduction caused by amorphization.

## 2 Materials and methods

### 2.1 Materials synthesis

The amorphous SSEs of binary *x*Li_2_S-(100-*x*)LiI (*x* = 10, 30, 50, 70, 90) were synthesized through a ball milling process. First, the starting materials of Li_2_S (Alfa Aesar, 99.9%) and LiI (Energy chemical, 98%) were ground in an agate mortar for 30 min to get the homogeneous mixture. Then, the stoichiometric mixtures of Li_2_S and LiI were ball-milled at 500 rpm for 33 h in a grinding jar with ZrO_2_ balls using planetary ball mill (Pulverisette 7 PL, Fritsch). The ball-to-powder mass ratio is 20:1 during sample preparation, and each cycle running for 15 min and resting for 5 min. The entire preparation process were carried out under an argon atmosphere (O_2_ < 0.1 ppm, H_2_O < 0.1 ppm).

### 2.2 X-ray diffraction measurements

PXRD measurements were conducted at room temperature on an Empyrean diffractometer from Malvern Panalytical using Cu Kα (*λ* = 1.541,874 Å) and a Bragg–Brentano geometry, for identify the phases of *x*Li_2_S-(100-*x*)LiI binary system. PXRD data were collected with 2*θ* ranging from 20° to 90° at a scan rate of 0.14° s^−1^. Before measurements, each sample was placed on a zero-background sample holder in an Ar-filled glovebox and protected by a Kapton film for the hygroscopicity of *x*Li_2_S-(100-*x*)LiI.

### 2.3 Electrochemical impedance spectroscopy measurements

Ionic conductivities of *x*Li_2_S-(100-*x*)LiI binary system were obtained through EIS measurement. Powder samples of *x*Li_2_S-(100-*x*)LiI were cold pressed into pellets under 4 tons in an insulative mold, and the pellets were placed between two stainless steel rods served as blocking electrodes. EIS measurement was performed on electrochemical workstation analyzer (AUTOLAB M204) in a frequency range from 1 MHz to 1 Hz with an amplitude of 50 mV. Moreover, the Nyquist curves were fitted by equivalent circuit to obtain the bulk resistance and grain boundary resistance of *x*Li_2_S-(100-*x*)LiI SSEs.

## 3 Results and discussion

**FIGURE 1:**
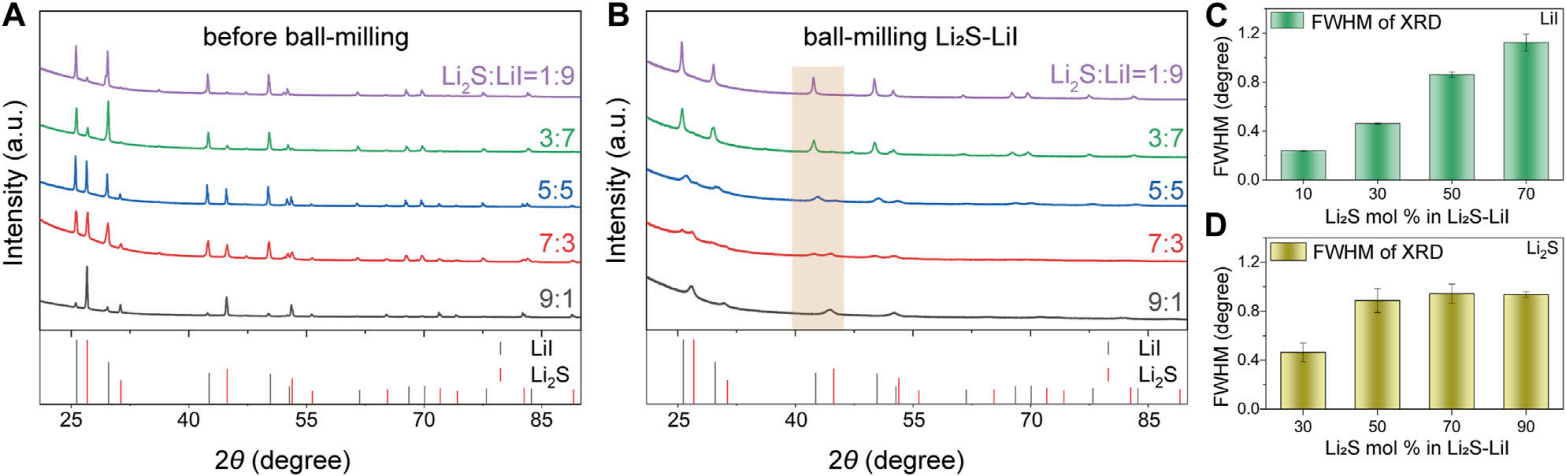
PXRD patterns of *x*Li_2_S-(100-*x*)LiI (*x* = 10, 30, 50, 70 and 90) **(A)** before ball-milling and **(B)** after ball-milling **(C)** FWHM of LiI peak in 40°–45° (*x* = 10, 30, 50, 70) **(D)** FWHM of Li_2_S peak in 40°–45° (*x* = 30, 50, 70, 90).

As presented in Figure 1, the amorphous degree of *x*Li_2_S-(100-*x*)LiI (*x* = 10, 30, 50, 70 and 90) system significantly depends on the stoichiometry of Li_2_S and LiI. Before ball-milling, all PXRD patterns of *x*Li_2_S-(100-*x*)LiI exhibit sharp-peak feature, which indicates their good crystallinity (Figure 1A). In contrast, the PXRD patterns of *x*Li_2_S-(100-*x*)LiI after ball-milling exhibit different degrees of broadening (Figure 1B). Representatively, FWHM of the PXRD peaks in the range of 40°–50° is used here to quantitatively analyze the amorphization degree of *x*Li_2_S-(100-*x*)LiI binary system (Indris et al., 2000; Sasano et al., 2011; Holder and Schaak, 2019; Londono-Restrepo et al., 2019; Schweiger et al., 2022; Sun et al., 2022). It should be emphasized that the peak positions and FWHM of Li_2_S or LiI at *x* = 10 or 90 are not discernible from the PXRD pattern due to the low content. Surprisingly, different stoichiometric ratios of Li_2_S and LiI in *x*Li_2_S-(100-*x*)LiI lead to obviously different amorphization degrees, even under the same ball-milling conditions. As shown in Figures 1C, D, the FWHM of LiI presents an increasing trend with the increase of Li_2_S and changes from 0.239 (*x* = 10) to 1.124 (*x* = 70), which demonstrates that the presence of Li_2_S can promote the amorphization of LiI. In contrast, the FWHM of Li_2_S seems to tend to remain constant as *x* increases in *x*Li_2_S-(100-*x*)LiI (*x* ≥ 50). Interestingly, the amorphization degree of the *x*Li_2_S-(100-*x*)P_2_S_5_ binary system is also dependent on the stoichiometric ratios of Li_2_S and P_2_S_5_ (Minami et al., 2006; Tatsumisago and Hayashi, 2012; Kudu et al., 2018). However, the amorphization degree of *x*Li_2_S-(100-*x*)P_2_S_5_ diminishes as Li_2_S increases, accompanied by the appearance of sharp peaks of Li_2_S in the PXRD patterns (Hayashi et al., 2004). Therefore, the difference between the *x*Li_2_S-(100-*x*)LiI and *x*Li_2_S-(100-*x*)P_2_S_5_ suggests that the amorphization degree depends not only on the stoichiometric ratios but also on the composition of the compound in the binary system.

**FIGURE 2:**
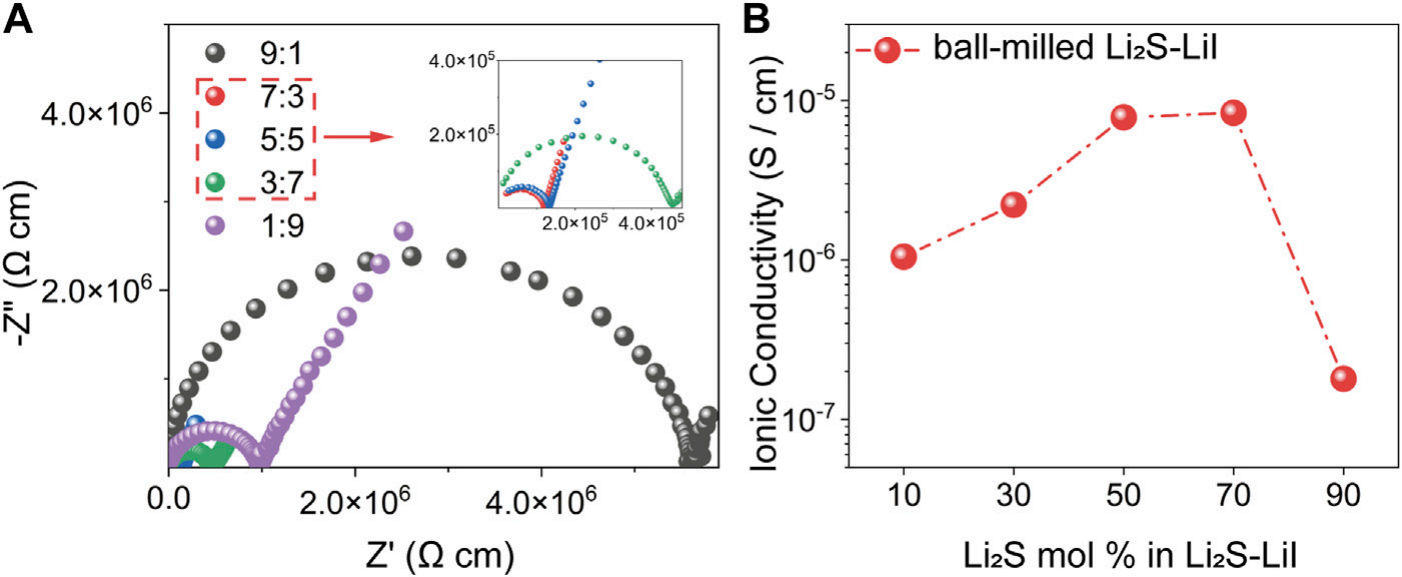
**(A)** Nyquist plots of the amorphous *x*Li_2_S-(10-*x*)LiI **(B)** Ionic conductivities of the amorphous *x*Li_2_S-(10-*x*)LiI.

The stoichiometric ratios of Li_2_S and LiI determine the amorphization degree of the *x*Li_2_S-(100-*x*)LiI binary system, which significantly affects its ionic conductivity. Figure 2A shows the Nyquist plots of amorphous *x*Li_2_S-(100-*x*)LiI binary system at room temperature (RT), and each curve exhibits a typical semicircle at high frequency representing the resistance and the linear part at low frequency representing ion blocking electrode. The EIS data were processed based on the formula: *Z* = (*Z*_0_ × *S*)/*l* to eliminate the effect of SSE pellet thickness and area on the impedance, in which *Z*_0_ is the raw data of the measured EIS, *l* is the thickness, and *S* is the area of SSE pellet. Fitting the plot by the equivalent circuit leads to the resistance *R*, which corresponds to the value of the real part of the Nyquist curve, and the ionic conductivity is calculated according to the formula of *σ* = *l*/(*R* × *S*). As presented in Figure 2B, the ionic conductivities of *x*Li_2_S-(100-*x*)LiI show a non-monotonic variation with the increase of *x*. As *x* increased from 10 to 70, the ionic conductivity of *x*Li_2_S-(100-*x*)LiI increased from 1.03 × 10^−6^ S/cm to 8.43 × 10^−6^ S/cm. Subsequently, after *x* continued to increase to 90, the ionic conductivity appeared to drop significantly to 1.78 × 10^−7^ S/cm. The above non-monotonic ionic conductivity changes may be attributed to both the amorphization degree of LiI and the content of Li_2_S in *x*Li_2_S-(100-*x*)LiI. In the first stage (*x* from 10 to 70), the amorphization of LiI is the dominant factor in influencing the ionic conductivity of *x*Li_2_S-(100-*x*)LiI (Figure 1C). However, in the next stage (*x* from 70 to 90), the adverse effect of Li_2_S content on ionic conductivity may play a major role.

**FIGURE 3:**
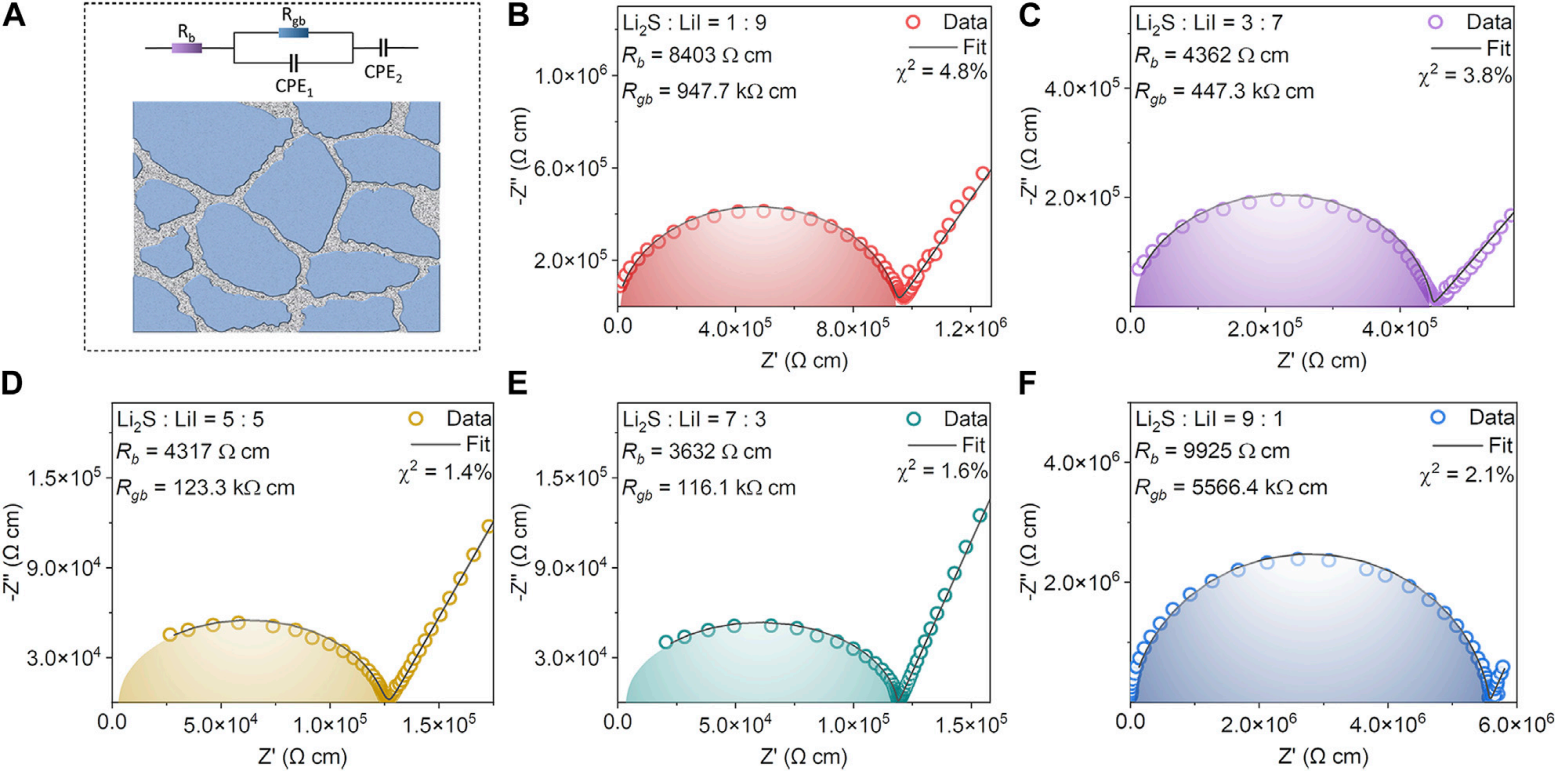
**(A)** Equivalent circuit model for SSEs, consisting of *R*_b_, *R*_gb_ and CPE. The fitting results of Nyquist plots of **(B)** 10Li_2_S-90LiI **(C)** 30Li_2_S-70LiI **(D)** 50Li_2_S-50LiI **(E)** 70Li_2_S-30LiI **(F)** 90Li_2_S-10LiI.

To understand the ionic transport mechanism of the amorphous *x*Li_2_S-(100-*x*)LiI in depth, the Nyquist plots were fitted with the equivalent circuit consisting of bulk resistance (*R*_b_), grain boundary resistance (*R*_gb_) and constant phase element (CPE). As illustrated in Figure 3A, lithium ions transport in the bulk phase and grain boundary of SSEs, which determines the overall ionic conductivity of *x*Li_2_S-(100-*x*)LiI (Gao et al., 2016; Goswami and Kant, 2019; Vadhva et al., 2021). Obviously, the hindrance of lithium ions transport at the grain boundaries is stronger than that of the bulk phase according to Figures 3B–F. For 10Li_2_S-90LiI, for example, its *R*_gb_ is 947.7 kΩ cm, which is much higher than that of *R*_b_ (8,403 *Ω* cm). Besides, the variation of *R*_gb_ is significantly higher than that of *R*_b_. The *R*_b_ and *R*_gb_ of 70Li_2_S-30LiI with the highest ionic conductivity are 3,632 *Ω* cm and 116.1 kΩ cm respectively. In contrast, the *R*_b_ and *R*_gb_ of 90Li_2_S-10LiI with the lowest ionic conductivity are 9,925 *Ω* cm and 5,566.4 kΩ cm respectively.

**FIGURE 4:**
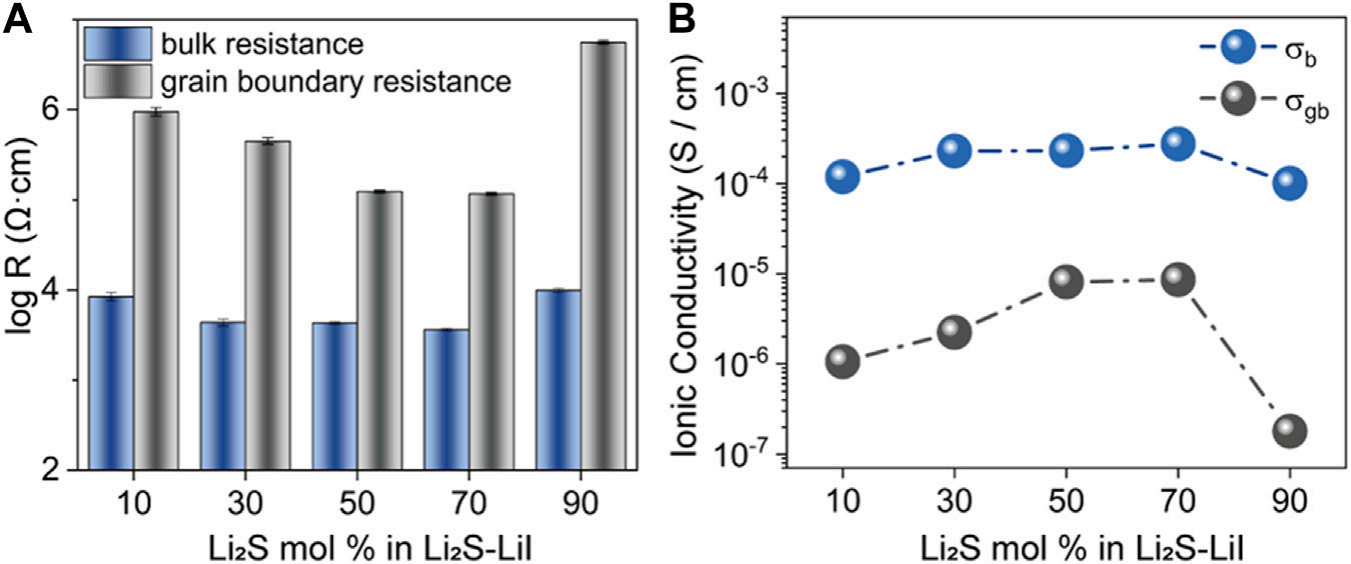
**(A)** Resistance of bulk and grain boundary in *x*Li_2_S-(100-*x*)LiI. **(B)** Ionic conductivity of bulk and grain boundary in *x*Li_2_S-(100-*x*)LiI.

**FIGURE 5:**
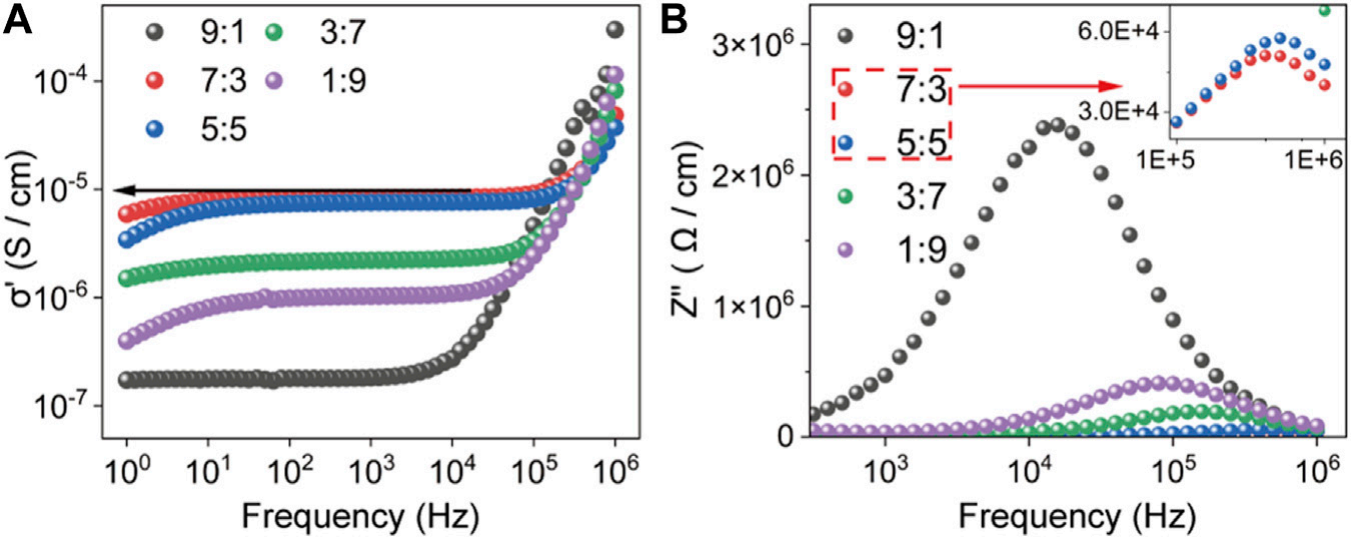
**(A)** The conductivity isotherms of *x*Li_2_S-(100-*x*)LiI **(B)** The imaginary part of the complex impedance of *x*Li_2_S-(100-*x*)LiI.

Furthermore, to present the dependence of *R*_b_ and *R*_gb_ on *x* in *x*Li_2_S-(100-*x*)LiI, the differences between *R*_b_ and *R*_gb_ on logarithmic scale are presented in Figure 4A. While the ionic conductivity of *x*Li_2_S-(100-*x*)LiI undergoes the significant change with *x* from 10 to 90 (Figure 2), *R*_b_ does not undergo a distinct fluctuation, as well as the bulk phase conductivity *σ*_b_. In contrast, *R*_gb_ and the grain boundary conductivity *σ*_gb_ show the significant changes and are in agreement with the trend of the ionic conductivity (Figure 4B). Also, the conductivity isotherms extracted from EIS can reflect the dependence of the grain boundary conductivity on *x* in *x*Li_2_S-(100-*x*)LiI, which is consistent with the results of the Nyquist curves fitted with the equivalent circuit. As shown in Figure 5A, conductivity isotherms are plotted from the real part (*σ*′) of the complex ionic conductivity as a function of frequency. Typically, the frequency independent plateaus (marked by arrow) correspond to the ionic conductivities at the grain boundary of SSEs (Schweiger et al., 2022). As *x* increases, the plateau of *σ*′ gradually reaches a maximum of 8.50 × 10^−6^ S/cm at *x* = 70, then dropping to a minimum of 1.79 × 10^−6^ S/cm at *x* = 90. It is worth emphasizing that the feature of conductivity isotherms not only agrees with the analysis of the Nyquist curve, but also the values corresponding to the plateau of *σ*′ are very close to the grain boundary conductivity *σ*_gb_ in Figure 4B, which confirms the above analysis of ionic conductivity of amorphous *x*Li_2_S-(100-*x*)LiI. In addition, the imaginary part (Z″) of the complex impedance as a function of frequency is plotted in Figure 5B, and the Z″ peak height is usually considered to be equal to half of the most resistive elements (here, i.e., the grain boundary resistance) in SSEs (Irvine et al., 1990). Consistently, the dependence of Z″ peak height on *x* can also corroborate the results of Nyquist curves fitted with the equivalent circuit.

Obviously, the above results indicate that the ionic conductivity change of amorphous *x*Li_2_S-(100-*x*)LiI depends directly on the grain boundary conductivity *σ*_gb_ and is almost unaffected by the bulk phase conductivity *σ*_b_. On the other hand, in combination with the PXRD data of *x*Li_2_S-(100-*x*)LiI (Figure 1), it can be concluded that the increase in grain boundary conductivity *σ*_gb_ may depend on the enhanced amorphization of LiI as *x* increases from 10 to 70, while the decrease in grain boundary conductivity *σ*_gb_ may be mainly affected by the increase in Li_2_S content as *x* increases from 70 to 90. In other words, there is a competitive relationship between the amorphization of LiI and the content of Li_2_S in affecting the grain boundary conductivity of amorphous *x*Li_2_S-(100-*x*)LiI.

## 4 Conclusion

In conclusion, the amorphous *x*Li_2_S-(100-*x*)LiI (10 ≤ *x* ≤ 90) binary system was synthesized by mechanical ball-milling method. The PXRD analysis significantly demonstrated that the increase of Li_2_S content can promote the amorphization of LiI, and the amorphous degree of Li_2_S tend to remain constant as *x* increases in *x*Li_2_S-(100-*x*)LiI (*x* ≥ 50). The EIS analysis revealed that the change in ionic conductivity of amorphous *x*Li_2_S-(100-*x*)LiI depends on the grain boundary conductivity and is almost unaffected by the bulk phase conductivity. In addition, the competitive mechanism between the amorphization of LiI and the content of Li_2_S in affecting the grain boundary conductivity was found. The findings of *x*Li_2_S-(100-*x*)LiI binary system provide insights into the future design of new amorphous SSEs.

## Data Availability

The raw data supporting the conclusion of this article will be made available by the authors, without undue reservation.
