# Supplementary material for: Effect of grain boundary resistance on the ionic conductivity of amorphous xLi2S-(100-x)LiI binary system
Source: Front Chem. 2023 Jul 20;11:1230187. doi: 10.3389/fchem.2023.1230187 (PMC10400117; doi:10.3389/fchem.2023.1230187)
Supplement: Supplementary file 1 [file DataSheet1.docx]

**Electronic Supplementary Information (ESI)**

**Effect of grain boundary resistance on the ionic conductivity of** **amorphous** ***x*Li_2_S-(100-*x*)LiI binary system**

Longbang Di,†^a^ Jiangyang Pan,†^a^ Lei Gao,*^b^ Jinlong Zhu,^a^ Liping Wang,^a^ Xiaomeng Wang,^a^ Qinqin Su,^a^ Song Gao,^b^ Ruqiang Zou,^b^ Yusheng Zhao,^c^ Songbai Han,^*a^

^a^Academy for Advanced Interdisciplinary Studies, Southern University of Science and Technology, Shenzhen 518055, China

^b^School of Materials Science and Engineering, Peking University, Beijing 100871, China

^c^Eastern Institute for Advanced Study, Ningbo 315201, China

Email : gaolei2018@pku.edu.cn

**Scanning electron microscope studies of morphology**

The morphology of *x*Li_2_S-(100-*x*)LiI SSE (*x* = 70) was characterized by SEM. As is shown in Figure S1, the SSE is mainly composed of particles with dimensions of approximately 2 μm. There are some differences in particle size, which may be caused by amorphous particle clusters.


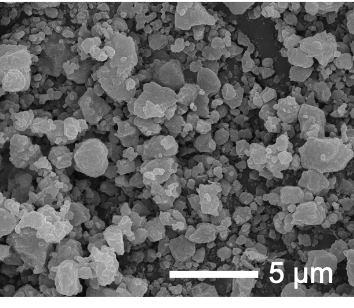


Figure S1. The SEM pattern of the 70Li_2_S-30LiI SSE.

**Raman measurements**


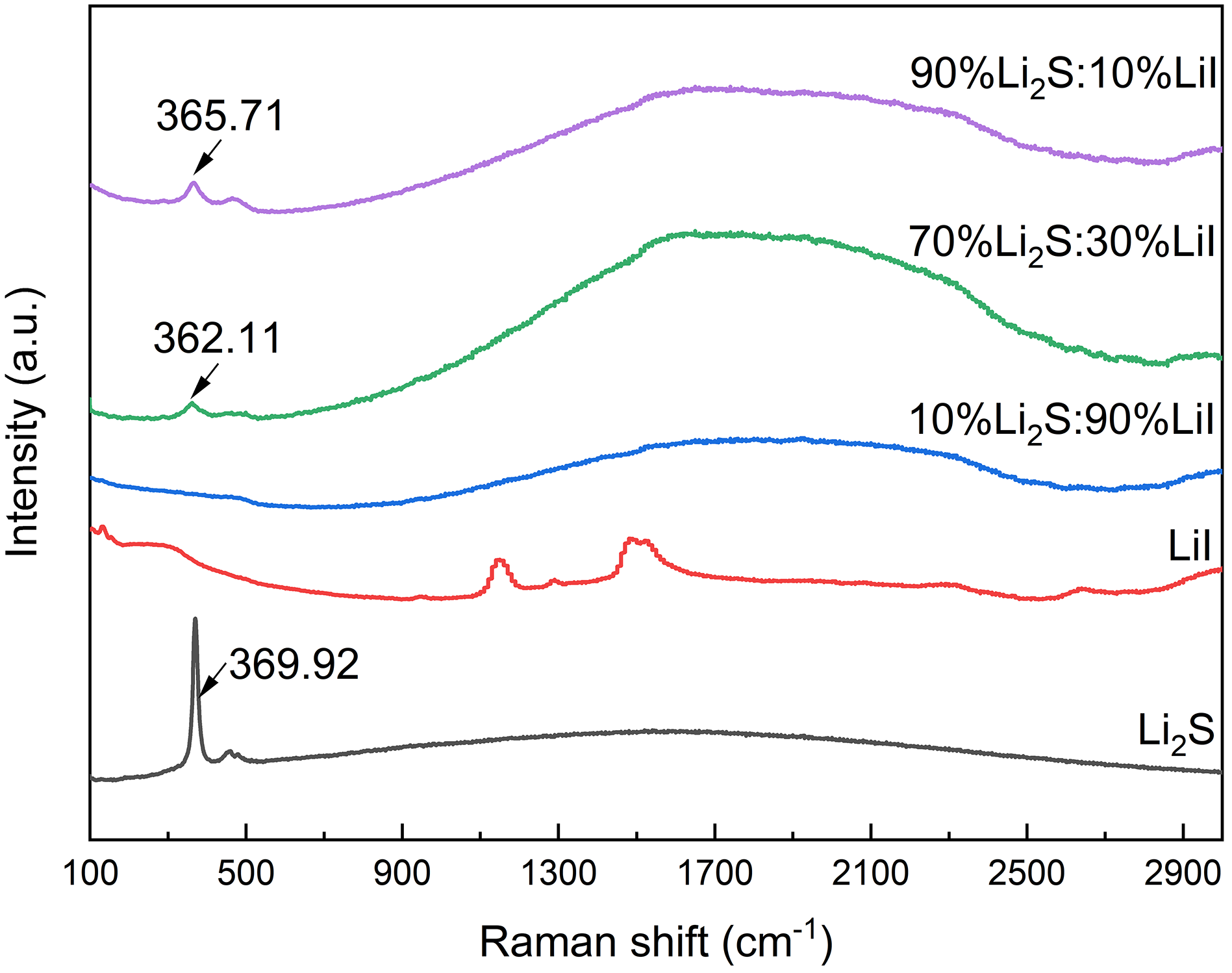


Figure S2. Obtained Raman spectra of Li_2_S, LiI and *x*Li_2_S-(100-*x*)LiI SSEs (x = 10, 70, 90).

Raman spectroscopy is used to determine if structural change has occurred in *x*Li_2_S-(100-*x*)LiI system. Three typical Raman spectra of *x*Li_2_S-(100-*x*)LiI SSEs (*x* = 10, 70, 90) are shown in Figure S2. The Raman peaks of *x*Li_2_S-(100-*x*)LiI SSEs (*x* = 70, 90) were observed at around 365 cm^-1^ and 470 cm^-1^, which correspond to the two Raman peaks of Li_2_S. Two interesting phenomena were observed. The first is that the Raman peak of LiI disappears in *x*Li_2_S-(100-*x*)LiI after ball-milling. The second is that the Raman peak of Li_2_S shifts to the left with increasing LiI content, which may be related to the increase in the Li-S bond length [1, 2]. To explain these interesting phenomena caused by amorphization, pair distribution function, neutron diffraction, nuclear magnetic resonance and other characterization methods will be used to conduct in-depth research on the local structure of *x*Li_2_S-(100-*x*)LiI system in the future.

**References**

[1] B. Choudhury, A. Choudhury, Dopant induced changes in structural and optical properties of Cr3+ doped TiO2 nanoparticles, Mater. Chem. Phys., 132 (2012) 1112-1118.

[2] L. Popović, D. de Waal, J.C.A. Boeyens, Correlation between Raman wavenumbers and PO bond lengths in crystalline inorganic phosphates, Journal of Raman Spectroscopy, 36 (2005) 2-11.
